# Familial Colorectal Cancer Type X in Central Iran: A New Clinicopathologic Description

**Published:** 2017-07-01

**Authors:** Mehrdad Zeinalian, Mahdi Hadian, Morteza Hashemzadeh-Chaleshtori, Rasoul Salehi, Mohammad Hassan Emami

**Affiliations:** 1 Department of Genetics and Molecular Biology, School of Medicine, Isfahan University of Medical Sciences, Isfahan, Iran; 2 Cellular and Molecular Research Center, Shahrekord University of Medical Sciences, Shahrekord, Iran; 3 Poursina Hakim Research Institute, Isfahan, Iran; 4 Department of Internal Medicine, School of Medicine, Isfahan University of Medical Sciences, Isfahan, Iran

**Keywords:** Familial colorectal cancer type X, Clinicopathologic, Lynch syndrome, Iran

## Abstract

**Background: **Familial colorectal cancer type X (FCCX) is a subtype of mismatch repair (MMR)-proficient colorectal cancerin which the patients are clinically at risk for Lynch syndrome (LS), a common hereditary cancer predisposing syndrome. In this study, we described a new clinicopathological feature of the condition in central Iran.

**Materials and Methods: **We designed a descriptive, retrospective study to screenat-risk colorectal cancer (CRC) patients, using Amsterdam II criteria and Molecular analysis in Isfahan (central Iran) throughout 2000-2013 period.

**Results:** 219 early-onset (≤ 50 years) CRC patients of 1659 were selected for the evaluation. Amsterdam II criteria were positive in 45 families; of whom 31 were finally analyzed by molecular testing.

MMR deficiency was detected in 7/31 probands (22.6%) as affected to LS, so 24 families (77.4%) were identified as FCCX. The mean age of the probands at diagnosis among FCCX families was 45.3 years (range 24-69) versus 38.0 years (range 31-50) in LS families. The frequency of CRC among FCCX and LS families was calculated 27.9% and 67.5%, respectively. Also, the most frequent extracolonic cancer among both FCCX and LS families was stomach by 25.5% and 30.8%, respectively. Tumor site was proximal to the splenic flexure in 20.8% and 57.1% of index CRC patients in FCCX and LS families, respectively.

**Conclusion: **Given the relative high frequency of FCCX and its different phenotype among Iranian populations, we need to set up more advanced molecular studies for exploration of unknown molecular pathways leading to tumorigenesis in this class of CRC patients.

## Introduction

 Colorectal cancer (CRC), the second cause of cancer-related mortality throughout the world ^1^^,^^3^ , presents hereditary pattern in at least 20% of the cases of which about 5% are related to inherited mutations in known cancer-predisposing genes and the rest has likely linked to unknown genetic changes ^4^^-^^6^ .

Hereditary nonpolyposis colorectal cancer (HNPCC) is used to describe a familial cluster of CRC with non-polyposis phenotype. At first, the Amsterdam criteria (AC) were introduced to screen CRC patients at risk for HNPCC according to a positive family history with at least three affected family members in two or more generations, and one being a first-degree relative of the other two and at least one individual diagnosed before 50 years of age^   7 ^. The AC1 refers to families with at least three colorectal cancers, while in AC2Lynch-associated extracolonic cancers containing cancers of endometrium, upper urinary tract, small bowel, stomach, liver, brain, breast, and skin were also included ^8^^,^^9^ . 

The HNPCC families are clinically heterogeneous of which about 4% are linked to Lynch syndrome (LS),which may be negative for AC, <1% are attributed to a Lynch-like syndrome, and 2–4% are defined as familial colorectal cancer type X (FCC-X)^10^^,^^11^. LS is identified by germline mutations in at least one of the mismatch-repair (MMR) genes including: MLH1, MSH2, MSH6, and PMS2, however, just about one-third of the LS families fulfill the AC criteria^12^^-^^15^. Although, Lynch-like syndrome (LLS) clinically demonstrates AC and tumors with functional MMR gene defects according to immunohistochemical staining and/or microsatellite instability (MSI) testing, no MMR gene mutations are found to justify the disease ^16^^,^^18^ . FCC-X families are AC positive families with MMR-proficient tumors and no MMR gene mutations^12^^, ^^19^^, ^^20^. 

According to recent studies, it seems there is a high frequency of FCC among Iranian populations^2^^,^^9^^, ^^21^^-^^23^.Meanwhile, no studies have been so far reported to describe clinicopathologic feature of FCC-X among our populations. Accordingly, we try to report a new description of FCC-X families in Central Iran.

## MATERIALS AND METHODS

 We undertook a descriptive retrospective study to screen FCC patients at risk for LS in Isfahan, Central Province of Iran. Of 1659 CRC patients registered in Poursina Hakim Research Center (PHRC), a referral gastrointestinal cancer center in Isfahan Province, in the 14-year period (2000-2013),at first all patients aged ≤ 50 years were included in our study as the early-onset patients. Then, all CRC patients with Amsterdam II criteria and their families were invited for genetic counseling through which the participants were interviewed about cancer-related family history at-least up to three generations. Amsterdam II criteria for primary clinical screening in our study included: having at least 3 affected members with one of the HNPCC-associated cancers (CRC, other GI cancers, endometrial, renal, breast (according to some resources), brain, skin, and pelvic cancers) in at least two successive generations, and being one of these three members a first-degree relative of the other two and at least one diagnosed before the age of 50 years.

The drawn pedigrees were reconfirmed by at-least two other members of every family. Besides, the cancer pathologic reports related to all affected family members were possibly requested to confirm the diagnosis, if available. Then, all resected tumors related to the index CRC patients were analyzed molecularly by both MSI testing and Immunohistochemistry (IHC) to detect probable MMR deficiency.

To detect MSI, a commercial pentaplex panel from Promega (MSI Analysis System, Version 1.2) including five mononucleotide markers (BAT-25, BAT-26, MON0-27,NR-21, and NR-24) was used, and two pentanucleotide markers (PentaC and Penta D)were used as indicators to detect specimen mix-ups. We considered tumors as MSI-High (MSI-H) if at least two of five quasi mononucleotide markers showed instability and MSI-Low (MSI-L) if only one marker was unstable.

For IHC, we used a formalin-fixed paraffin-embedded (FFPE) tissue block for each case, including both tumoral and adjacent healthy mucosa. After providing at least a proper section for each protein, we treated the slides with primary antibodies related to MMR proteins according to IHC guideline specific for each immunologic product. The properties of the antibodies were as following: MSH2 (Leica Biosystems: Novocastra, UK, Lyophilized, Product Code (PC): NCL-MSH2) at 1/80 dilution, MLH1 (Leica Biosystems: Novocastra, UK, Liquid, PC: NCL-L-MLH1) at 1/100 dilution, MSH6 (Leica Biosystems: Novocastra, UK, Liquid, PC: NCL-L-MSH6) at 1/100 dilution, and PMS2 (Leica Biosystems: Novocastra, UK, Liquid, PC: NCL-L-PMS2) at 1/100 dilution. Then, the slides were incubated with Post Primary Block reagent and DAB working solution for some minutes, respectively. If the MMR protein has been expressed, the nuclear staining will be present. MMR deficiency leads to absent nuclear staining in tumor section compared to normal adjacent tissue.

Data were analyzed using SPSS statistics software (Ver.19).

**Ethical approval**

Ethical approval was received from the Medical Ethics Committee of Shahrekord University of Medical Sciences (Research project Number: 003). The research was carried out according to principles set out in the Declaration of Helsinki 1964 and all subsequent revisions. Informed consent was obtained, and the privacy and confidentiality were observed throughout the study.

## Results

 We finally identified that 413/1659 (24.9%) CRC patients registered in PHRC throughout 2000-2013 period were affected by early-onset (age ≤ 50 years at diagnosis) CRC. Family history was positive in 72 / 219 patients responding probands (32.9%) and among whom we found finally 53 families (24.2%) with at least three members affected by any type of Lynch-associated cancer according to Amsterdam II criteria. We classified them as “Familial Colorectal Cancer” (FCC) families. Using Amsterdam II criteria, 45/53FCC families were clinically identified as HNPCC families and were considered candidates for molecular testing. The 14 probands were excluded due to lack of their tumor tissues or being unwilling to incorporate.

After both MSI testing and IHC-MMRs, 7 index CRC patients were identified as MMR-deficient group (22.6%), while no MMR deficiency was finally detected among the rest of 24 probands and they were classified as “FCC-X” patients (77.4%).

Mean age at diagnosis in MMR-deficient probands was 38.0 years (range 31-50) versus 45.3 years (range 24-50) (P value < 0.05) among FCC-X probands.

Among MMR-deficient probands, altogether, 57.2% of tumors were located in right colon (including cecum, ascending colon, and transverse colon), while among FCC-X probands 20.8% of tumors were located in right colon. 

**Figure 1:**
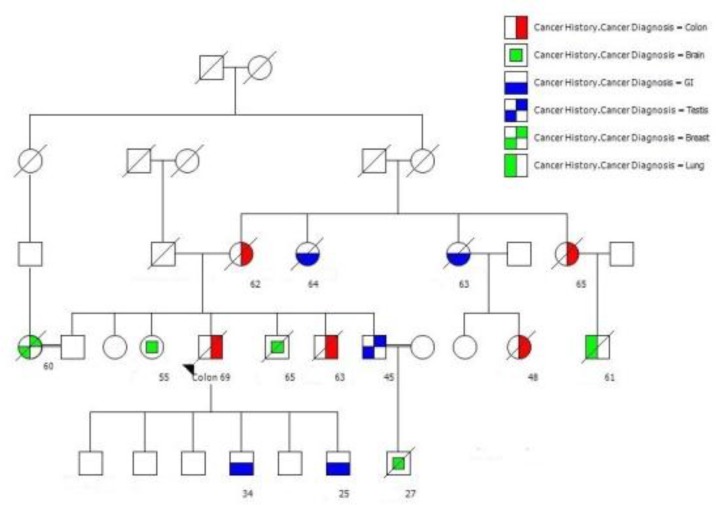
A large pedigree with at least 15 affected members with 6 different cancers. Note that the age onset of cancer is being earlier in recent generation

**Figure 2:**
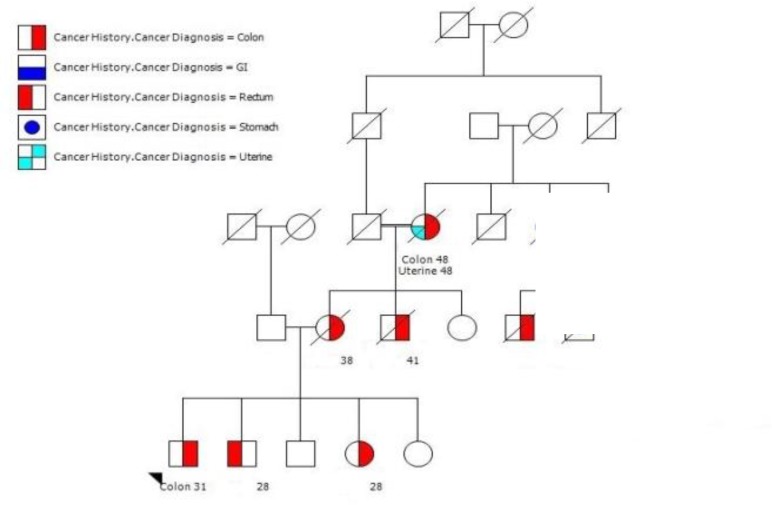
A large pedigree with at least 10 affected members with 5 different cancers. Note that the age onset of cancer is being earlier in recent generation

History of cancer was found in 186 members within 31 HNPCC families. Of whom, 140 cancer patients were related to 24 FCC-X families (5.8 patients per family) and 46 cancer patients were belonged to 7 MMR-deficient families (6.6 patients per family) (Figures 1, 2).

Age at diagnosis of cancer was averagely 51.7 and 51.0 among FCC-X families and MMR-deficient families, respectively (P-value = 0.817).

Colorectal, stomach, and hematopoietic system with 67.4, 8.7, and 6.5 percent, respectively were most frequent cancers among MMR-deficient families, while among FCC-X families, colorectal, stomach and lung with 39.3, 10.0, and 8.6 percent, respectively, were most frequent cancers (Table 1).

**Table 1:** Cancer sites among Iranian families with familial colorectal cancers in both FCC-X and MMR deficient subsets

**Cancer site**	**FCC-X families**		**MMR deficient ** **families**		**Sum**
	**Frequency**	**Percent**	**Frequency**	**Percent**	
Colorectal	55	39.3	31	67.4	86
Stomach	14	10.0	4	8.7	18
Lung	12	8.6	2	4.3	14
Breast	11	7.9	1	2.2	12
Brain	9	6.4	0	0.0	9
Hepatobiliary tract	7	5.0	2	4.3	9
Intestine	6	4.3	0	0.0	6
Prostate	4	2.9	2	4.3	6
Uterus	4	2.9	1	2.2	5
Skin	3	2.1	0	0.0	3
Hematopoietic system	3	2.1	3	6.5	6
Bladder	3	2.1	0	0.0	3
Thyroid	2	1.4	0	0.0	2
Testis	2	1.4	0	0.0	2
Bone	2	1.4	0	0.0	2
Kidney	1	0.7	0	0.0	1
Pancreas	1	0.7	0	0.0	1
Nasopharynx	1	0.7	0	0.0	1
Total	140	100	46	100	186

FCC-X: Familial colorectal cancer type X; MMR: Mismatch-repair

Pathologically, 8/24 of FCC-X probands (33.3%) were diagnosed at early stages (I or II pathologic TNM stage). In MMR-deficient probands, however, 1/7 (14.3%) cases were diagnosed at early stages Moreover, mortality rate among FCC-X probands and MMR-deficient probands was 11/24(45.8%) and 1/7 (14.3%), respectively in our study.

## Discussion

 This paper is the first description of FCC-X among Iranian patients. Given the absence of any systematic program for screening and early detection of at-risk Iranian families, we tried to set up a new pilot study to explore clinicohistopathologic and epidemiologic features of familial colorectal cancer in central Iran. Accordingly, we applied AC-II to screen CRC patients at risk for Lynch syndrome. 

More than 74% of our Amsterdam positive probands were MMR-proficient, determining a likely high prevalence of FCC-X among our population. According to different studies, particularly among western countries, averagely 21-73% of CRC patients with clinical AC-I/AC-II criteria represent finally FCC-X^12^^,^^24^^-^^26^. Apparently, the frequency of FCC-X among Iranian CRC patients is more than western countries. So, more molecular evaluations are necessary to explore genetic causes of this common subtype of CRC among our population.

**Distinct Clinicopathologic Phenotype**

Our FCC-X probands were diagnosed averagely more than 7 years later than MMR-deficient probands. It is similar to other studies around the world, so according to a review article, it was calculated 57.3 years in FCC-X versus 49.7 in Lynch syndrome ^27^^-^^30^ . Moreover, a majority of FCC-X CRC tumors were found in left colon (79.2% vs. 42.8% in MMR-deficient tumors). It has been also presented in many similar studies among other populations according which 70% of FCC-X CRC have been found in left side colon^12^^,^^30^^-^^32^ .

Although there was no meaningful difference between FCC-X and MMR-deficient families in case of mean age at diagnosis and the average number of cancer patients (rather than probands), extracolonic cancers in FCC-X families included more spectrum than MMR-deficient ones. So, altogether 18 organs were affected in cancer patients among FCC-X families versus 8 organs in MMR-deficient families. This finding is against the results obtained from similar studies among other non-Iranian populations, so according to some of the findings, the risk of extracolonic cancers is increased in Lynch syndrome compared to FCC-X families ^33^^-^^35^ .

Although a more proportion of FCC-X probands in comparison to MMR-deficient ones was identified in early pathologic stage (33.3% vs. 14.3%), mortality rate of FCC-X probands was calculated higher than MMR-deficient ones (45.8% vs.14.3%). It could be due to a relative better survival in MMR- deficient CRC patients in comparison to FCC-X CRC patients, an issue for which there is much evidence in worldwide studies^12^^,^^34^^-^^37^.

Distinct clinical phenotype of FCC-X tumors in comparison to MMR-deficient ones is suggestive of a different molecular basis for this common subtype of CRC about which more evaluations are necessary. It also proposes a different protocol for screening and early detection of cancer among FCC-X families. Therefore, given the high frequency of FCC-X among Iranian population, setting up a native screening program according to different properties of all CRC subtypes must be considered. 

## CONCLUSION

 Although there is not enough data about clinical, molecular, and histopathological features of familial colorectal cancer among Iranian populations, apparently FCC-X subset of this group of cancers is more frequent in our population. Distinct clinical and histopathological phenotype of FCC-X is suggestive of new molecular mechanisms. It also underscores the necessity of revision in guidelines for screening and early-detection of cancer among FCC-X families.
